# Oral consultation needs and diagnostic patterns by psychiatric disorder subtype among psychiatric inpatients in a general hospital: a retrospective study

**DOI:** 10.1186/s12903-026-08784-8

**Published:** 2026-06-02

**Authors:** Ling Tian, Yiqing Lan, Shanshan Chu, Wei Liu, Jiahui Wen, Xu Fang

**Affiliations:** Tongde Hospital of Zhejiang Province Affiliated to Zhejiang Chinese Medical University (College of Integrated Traditional Chinese and Western Medicine Clinical Medicine, Hangzhou, China

**Keywords:** Mental disorders, Psychiatric inpatients, Oral consultation, Tooth loss, Retrospective study

## Abstract

**Background:**

Evidence on oral consultation needs and diagnostic patterns among psychiatric inpatients remains limited. This study compared oral consultation needs and diagnostic patterns across psychiatric disorder subtypes in a general hospital and examined the effects of age and sex on related outcomes.

**Methods:**

This retrospective study included oral consultation records of psychiatric inpatients who received oral consultations during hospitalization at Tongde Hospital of Zhejiang Province Affiliated to Zhejiang Chinese Medical University between January 2022 and December 2025. Age, sex, psychiatric disorder subtype, consultation demand, and oral diagnosis were extracted. Differences across subtypes were assessed using Fisher’s exact test with Monte Carlo simulation, and Cramér’s V was calculated as a descriptive measure of effect size. Logistic regression was performed using consultation demand for prosthetic treatment of missing teeth as the outcome, with adjustment for sex and age quartiles; sensitivity analysis modeled age as a continuous variable.

**Results:**

A total of 436 psychiatric inpatients were included. Tooth pain was the most common consultation demand (42.43%). Consultation demands and oral diagnostic distributions differed significantly across psychiatric disorder subtypes (Monte Carlo Fisher’s exact *P* < 0.001 for both; Cramér’s V = 0.195 and 0.210, respectively). Patients with organic mental disorders showed a higher proportion of consultation demand for prosthetic treatment of missing teeth, whereas patients with schizophrenia showed higher proportions of tooth pain and residual roots/crowns. In the primary quartile-based multivariable model, organic mental disorders were associated with consultation demand for prosthetic treatment of missing teeth (aOR = 2.11, 95% CI: 1.08–4.13, *P =* 0.029), and age was consistently associated with this outcome across the main and sensitivity analyses.

**Conclusions:**

Oral consultation needs and diagnostic patterns differed across psychiatric disorder subtypes among psychiatric inpatients who received oral consultations. Organic mental disorders were associated with consultation demand for prosthetic treatment of missing teeth in the primary model, while age showed a consistent association with this outcome. These findings describe oral consultation patterns rather than overall oral disease prevalence or differences from non-psychiatric hospitalized patients, and may help inform oral health assessment and referral strategies in psychiatric inpatient care.

**Supplementary Information:**

The online version contains supplementary material available at 10.1186/s12903-026-08784-8.

## Background

Mental disorders are a major global public health challenge and continue to place a substantial burden on healthcare systems and society. With increasing emphasis on the overall health management of people with mental disorders, greater attention has been paid to their physical health, among which oral health is particularly important. Previous studies have shown that patients with schizophrenia, mood disorders, and other severe mental illnesses often have poor oral hygiene and higher rates of dental caries, periodontal disease, and tooth loss, while oral pain and impaired mastication are also common [1, 2]. Oral problems may not only compromise nutritional intake and quality of life, but may also aggravate psychiatric symptoms, reduce treatment adherence, and increase the complexity of inpatient management [3].

The risk of oral health problems may be even greater in the inpatient psychiatric setting of a general hospital. Hospitalized patients often present with cognitive impairment, behavioral disturbances, complex psychotropic medication use, and reduced self-care ability, all of which may contribute to the accumulation or progression of oral disease [4, 5]. However, most existing studies have focused on the prevalence of oral diseases in people with mental disorders, while relatively little attention has been given to oral consultation patterns and diagnostic profiles during psychiatric hospitalization [6]. Whether oral consultation needs and diagnostic patterns differ across psychiatric disorder subtypes remains insufficiently explored. Clarifying these differences may help improve the early recognition of oral problems and facilitate timely dental referral among psychiatric inpatients [7, 8].

Tongde Hospital of Zhejiang Province, Affiliated to Zhejiang Chinese Medical University, has an independent psychiatric inpatient campus and a large psychiatric inpatient population, providing a stable clinical data source for the systematic evaluation of oral consultation needs in this setting. Against this background, the present study used oral consultation records from psychiatric inpatients in a general hospital to characterize the consultation needs and diagnostic distributions among patients who received oral consultations during hospitalization, to compare these patterns across psychiatric disorder subtypes, and to examine factors associated with consultation demand for prosthetic treatment of missing teeth among psychiatric inpatients who received oral consultations.

## Methods

### Study design and setting

This was a single-center retrospective study. The study population was drawn from patients hospitalized in the psychiatric department of Tongde Hospital of Zhejiang Province Affiliated to Zhejiang Chinese Medical University between January 2022 and December 2025. The hospital is a general hospital with an independent psychiatric inpatient campus and a stable source of psychiatric inpatients. Using the hospital information system and oral consultation records, we analyzed psychiatric inpatients who received oral consultations during hospitalization to characterize their oral consultation needs and diagnostic patterns. This study was approved by the Ethics Committee of Tongde Hospital of Zhejiang Province Affiliated to Zhejiang Chinese Medical University (approval no. R20260012).

### Participants

We included patients who were hospitalized for mental disorders in the psychiatric department of Tongde Hospital of Zhejiang Province Affiliated to Zhejiang Chinese Medical University between January 2022 and December 2025 and who received oral consultations during hospitalization. For patients with multiple oral consultations during the same admission, only the first consultation record was included in the analysis.

The inclusion criteria were as follows:

- admission to the psychiatric department of Tongde Hospital of Zhejiang Province Affiliated to Zhejiang Chinese Medical University during the study period, with a primary diagnosis of a mental disorder;
- a complete oral consultation record during hospitalization; and.
- complete key information, including age, sex, psychiatric disorder subtype, consultation demand, and oral diagnosis.

The exclusion criteria were as follows:

- missing key information that could not be verified; and.
- consultation records without a clearly documented primary consultation demand or primary diagnosis.

A total of 436 patients were ultimately included.

### Data collection and variable definition

Patient data were extracted from the hospital information system and oral consultation records, including age, sex, psychiatric disorder subtype, consultation demand, and oral diagnosis. Psychiatric disorder subtype was classified according to the principal psychiatric diagnosis and coded using the International Classification of Diseases, 10th Revision (ICD-10). Based on the characteristics of the study population, psychiatric disorders were grouped into organic mental disorders, schizophrenia, mood disorders, anxiety disorders, and other mental disorders, the latter including dissociative/conversion disorders, personality disorders, anorexia nervosa, and childhood emotional disorders. In this study, organic mental disorders mainly included dementia-related disorders, delirium, and other mental disorders secondary to organic brain disease or systemic medical conditions, as defined by the principal ICD-10-coded psychiatric diagnosis recorded in the hospital records.

For patients with multiple oral consultations during the same admission, only the first consultation record was included in the analysis. Because the system recorded a single chief complaint and a single primary diagnosis for each consultation, both consultation demand and oral diagnosis were classified according to the primary item. This approach was adopted to ensure consistency of classification across records and to reflect the principal reason for referral and the principal oral condition documented in the consultation note. However, because secondary complaints and coexisting oral conditions were not systematically recorded in the study dataset, this approach may not fully capture the complexity of oral comorbidity, and the frequency of patients with multiple oral conditions could not be quantified. Consultation demands were categorized as tooth pain, prosthetic treatment of missing teeth, tooth mobility, oral mucosal discomfort, tooth defect, and other. Oral diagnoses were categorized as tooth loss, pulpitis/apical periodontitis, residual roots/residual crowns, periodontal disease, oral mucosal disease, hard tissue defect, and other. The main comparisons were the distributions of consultation demands and primary oral diagnoses across psychiatric disorder subtypes.

### Statistical analysis

Statistical analyses were performed using SPSS version 26.0. Continuous variables were expressed as mean ± standard deviation (mean ± SD), and categorical variables were presented as frequencies and percentages [n (%)]. Fisher’s exact test with Monte Carlo simulation based on 10,000 samples was used to assess differences in the distributions of consultation demands and oral diagnostic patterns across psychiatric disorder subtypes, because more than 20% of cells had expected counts < 5 in the contingency tables and the assumptions for the Pearson chi-square test were therefore not met. Because Fisher’s exact test does not yield χ² statistics or degrees of freedom, overall significance was reported using Monte Carlo Fisher’s exact P-values. Cramér’s V was reported as a descriptive measure of effect size based on the corresponding contingency tables. When the overall difference was statistically significant, pairwise comparisons were conducted using pairwise Fisher’s exact tests with Bonferroni correction to control for type I error due to multiple testing.

In the logistic regression analysis, age was categorized into quartiles based on the age distribution of the study population: Q1 (≤ 56 years), Q2 (> 56 to ≤ 68 years), Q3 (> 68 to ≤ 78.5 years), and Q4 (> 78.5 years). Based on the main clinical findings and variable distribution, consultation demand for prosthetic treatment of missing teeth (yes/no) was selected as the binary outcome variable. Logistic regression analysis was performed using the enter method rather than stepwise variable selection. Organic mental disorders were included as the main explanatory variable because this subgroup showed the highest proportion of consultation demand for prosthetic treatment of missing teeth in the descriptive analysis. Therefore, the regression analysis was intended to explore associated factors within the consulted inpatient population rather than to establish causal relationships. Sex and age quartiles were included as covariates based on clinical relevance and their potential confounding effects on tooth loss and prosthetic treatment needs. To assess the potential influence of age categorization on the estimated effect sizes, a sensitivity analysis was performed by modeling age as a continuous variable instead of age quartiles. Other potential confounders, including hospitalization duration, medication exposure, functional status, and oral care practices, were not included in the multivariable model because these variables were not available in the retrospective dataset. No automated variable selection procedure was used. Additional logistic regression models for less frequent consultation demand categories were not performed because of small subgroup sizes, sparse outcome events in some categories, and the exploratory nature of the study. Results were reported as odds ratios (ORs) or adjusted odds ratios (aORs) with 95% confidence intervals (95% CIs). All tests were two-sided, and *P <* 0.05 was considered statistically significant. No a priori sample size calculation was performed because this was a retrospective observational study based on existing clinical records.

## Results

### Baseline characteristics

A total of 436 psychiatric inpatients who received oral consultations were included, comprising 181 men (41.51%) and 255 women (58.49%). The mean age was 64.38 ± 19.07 years, with a range from 12 to 104 years. The psychiatric disorder subtypes were distributed as follows: organic mental disorders, 165 cases (37.84%); schizophrenia, 144 cases (33.03%); mood disorders, 82 cases (18.81%); anxiety disorders, 17 cases (3.90%); and other disorders, 28 cases (6.42%). Among all consultation demands, tooth pain was the most common (42.43%), followed by prosthetic treatment of missing teeth (29.59%). Among oral diagnoses, tooth loss accounted for the highest proportion (28.44%), followed by pulpitis/apical periodontitis (19.95%) and residual roots/residual crowns (19.50%) (Table 1).

**Table 1:** Baseline characteristics of psychiatric inpatients receiving oral consultations (*n =* 436)

Variable	*n* (%)
Age, years	64.38 ± 19.07
Sex	
Male	181 (41.51)
Female	255 (58.49)
Psychiatric disorder subtype	
Organic mental disorders	165 (37.84)
Schizophrenia	144 (33.03)
Mood disorders	82 (18.81)
Anxiety disorders	17 (3.90)
Other	28 (6.42)
Consultation demand	
Tooth pain	185 (42.43)
Prosthetic treatment of missing teeth	129 (29.59)
Tooth mobility	41 (9.40)
Oral mucosal discomfort	36 (8.26)
Tooth defect	19 (4.36)
Other	26 (5.96)
Oral diagnosis	
Tooth loss	124 (28.44)
Pulpitis/apical periodontitis	87 (19.95)
Residual roots/residual crowns	85 (19.50)
Periodontal disease	63 (14.45)
Oral mucosal disease	26 (5.96)
Hard tissue defect	24 (5.50)
Other	27 (6.19)

Age is presented as mean ± standard deviation (SD). All other variables are presented as *n* (%)

Baseline characteristics stratified by psychiatric disorder subtype are shown in Table 2. Patients with organic mental disorders were older than those in the other psychiatric disorder subgroups, with a mean age of 79.15 ± 11.27 years, whereas patients in the other disorders group had the lowest mean age of 46.14 ± 21.97 years. The proportion of female patients was highest in the organic mental disorders group (73.94%), whereas male patients accounted for a slightly higher proportion in the schizophrenia and mood disorder groups. Most patients were able to cooperate with treatment across all psychiatric disorder subtypes.

**Table 2:** Baseline characteristics of psychiatric inpatients receiving oral consultations by psychiatric disorder subtype

Variable	Overall (*n =* 436)	Organic (*n =* 165)	Schizophrenia (*n =* 144)	Mood (*n =* 82)	Anxiety (*n =* 17)	Other (*n =* 28)
Age, years	64.38 ± 19.07	79.15 ± 11.27	59.33 ± 12.04	51.11 ± 21.28	58.00 ± 13.21	46.14 ± 21.97
Male	181 (41.51)	43 (26.06)	77 (53.47)	44 (53.66)	6 (35.29)	11 (39.29)
Female	255 (58.49)	122 (73.94)	67 (46.53)	38 (46.34)	11 (64.71)	17 (60.71)
Cooperative	426 (97.71)	164 (99.39)	138 (95.83)	80 (97.56)	17 (100.00)	27 (96.43)
Uncooperative	10 (2.29)	1 (0.61)	6 (4.17)	2 (2.44)	0 (0.00)	1 (3.57)

Age is presented as mean ± standard deviation (SD); categorical variables are presented as n (%). Abbreviations: Organic, organic mental disorders; Mood, mood disorders; Anxiety, anxiety disorders; Other, other psychiatric disorders

### Consultation needs

**Fig. 1:**
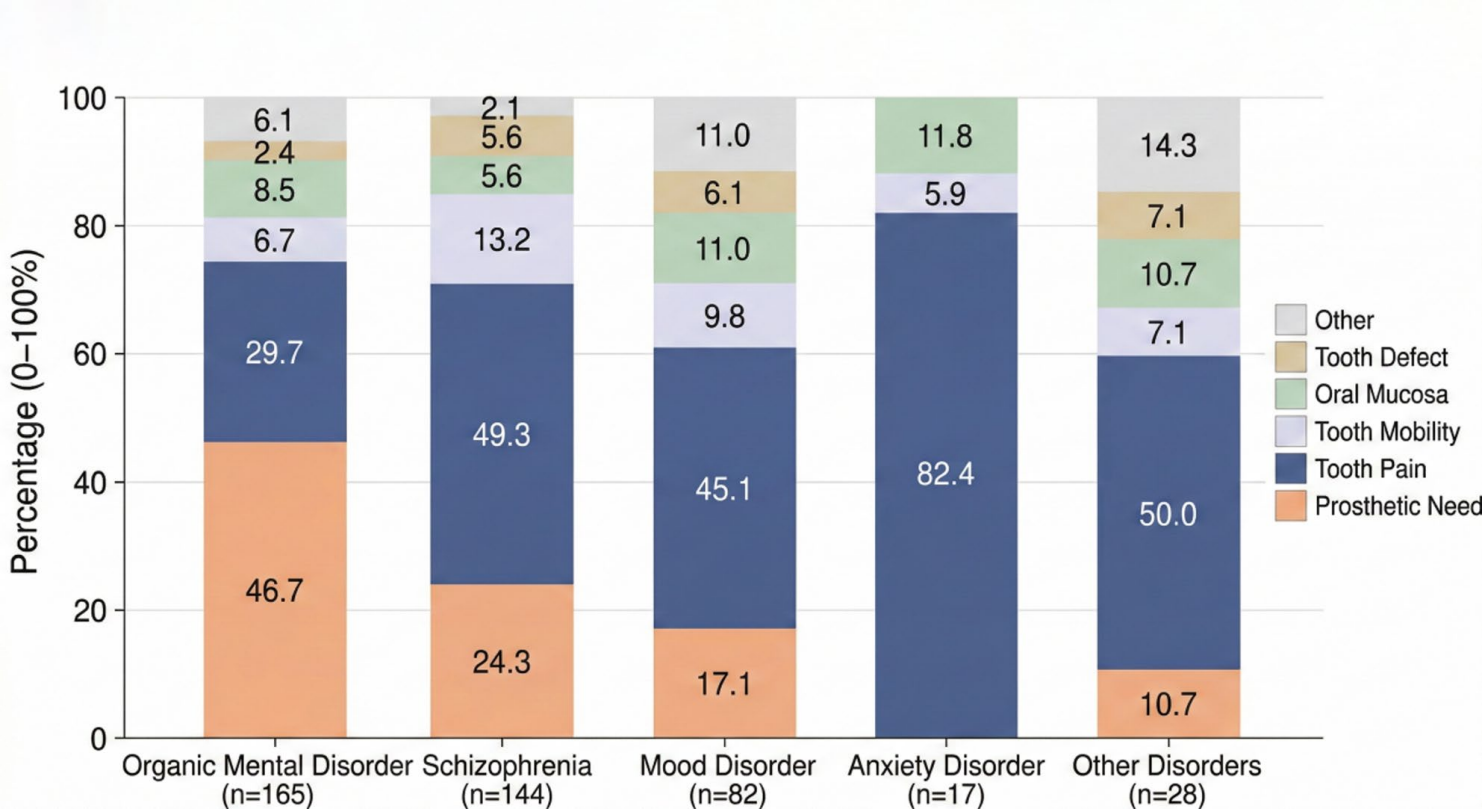
Visual summary of oral consultation demand distributions across psychiatric disorder subtypes

The distribution of consultation demands differed significantly across psychiatric disorder subtypes (Monte Carlo Fisher’s exact *P* < 0.001; Cramér’s V = 0.195), indicating an association. Pairwise comparisons showed that consultation demand for prosthetic treatment of missing teeth was more frequent in patients with organic mental disorders than in all other psychiatric disorder subtypes. Tooth pain accounted for a high proportion of consultations in patients with anxiety disorders and schizophrenia. Tooth mobility, oral mucosal discomfort, and tooth defect did not differ significantly across psychiatric disorder subtypes. Detailed pairwise comparisons are provided in Supplementary Table S1. The proportional distribution of consultation demands across psychiatric disorder subtypes is shown in Fig. 1; Table 3.

**Table 3:** Distribution of oral consultation demands across psychiatric disorder subtypes

Consultation demand	Organic mental disorders (*n =* 165)	Schizophrenia (*n =* 144)	Mood disorders (*n =* 82)	Anxiety disorders (*n =* 17)	Other (*n =* 28)
Tooth pain	49 (29.70)	71 (49.31)	37 (45.12)	14 (82.35)	14 (50.00)
Prosthetic treatment of missing teeth	77 (46.67)	35 (24.31)	14 (17.07)	0 (0.00)	3 (10.71)
Tooth mobility	11 (6.67)	19 (13.19)	8 (9.76)	1 (5.88)	2 (7.14)
Oral mucosal discomfort	14 (8.48)	8 (5.56)	9 (10.98)	2 (11.76)	3 (10.71)
Tooth defect	4 (2.42)	8 (5.56)	5 (6.10)	0 (0.00)	2 (7.14)
Other	10 (6.06)	3 (2.08)	9 (10.98)	0 (0.00)	4 (14.29)

Data are presented as n (%). Differences in oral consultation demand distributions across psychiatric disorder subtypes were assessed using Fisher’s exact test with Monte Carlo simulation based on 10,000 samples. The overall difference was statistically significant (Monte Carlo Fisher’s exact *P* < 0.001; Cramér’s V = 0.195). Pairwise comparisons were performed using Fisher’s exact test with Bonferroni correction, and detailed results are provided in Supplementary Table S1

Percentage distribution of oral consultation demands among different psychiatric disorder subtypes. Each stacked bar represents 100% of consultation demands within each subgroup. Exact numbers and percentages are provided in Table 3.

### Diagnostic patterns

The distribution of oral diagnostic patterns differed significantly across psychiatric disorder subtypes (Monte Carlo Fisher’s exact *P* < 0.001; Cramér’s V = 0.210), indicating an association. Pairwise comparisons showed that tooth loss was more frequent in patients with organic mental disorders than in all other psychiatric disorder subtypes, while residual roots/residual crowns were more frequent in patients with schizophrenia than in patients with organic mental disorders. Pulpitis/apical periodontitis was less frequent in patients with organic mental disorders than in mood disorders, anxiety disorders, and other psychiatric disorders. Detailed pairwise comparisons are provided in Supplementary Table S2. The distribution of oral diagnoses across psychiatric disorder subtypes is shown in Table 4.

**Table 4:** Distribution of oral diagnoses across psychiatric disorder subtypes

Oral diagnosis	Organic mental disorders (*n =* 165)	Schizophrenia (*n =* 144)	Mood disorders (*n =* 82)	Anxiety disorders (*n =* 17)	Other (*n =* 28)
Tooth loss	76 (46.06)	32 (22.22)	14 (17.07)	0 (0.00)	2 (7.14)
Residual roots/residual crowns	21 (12.73)	40 (27.78)	15 (18.29)	4 (23.53)	5 (17.86)
Pulpitis/apical periodontitis	20 (12.12)	27 (18.75)	23 (28.05)	7 (41.18)	10 (35.71)
Periodontal disease	23 (13.94)	25 (17.36)	8 (9.76)	4 (23.53)	3 (10.71)
Oral mucosal disease	10 (6.06)	6 (4.17)	7 (8.54)	1 (5.88)	2 (7.14)
Hard tissue defect	4 (2.42)	11 (7.64)	6 (7.32)	1 (5.88)	2 (7.14)
Other	11 (6.67)	3 (2.08)	9 (10.98)	0 (0.00)	4 (14.29)

Data are presented as n (%). Differences in oral diagnostic distributions across psychiatric disorder subtypes were assessed using Fisher’s exact test with Monte Carlo simulation based on 10,000 samples. The overall difference was statistically significant (Monte Carlo Fisher’s exact *P* < 0.001; Cramér’s V = 0.210). Pairwise comparisons were performed using Fisher’s exact test with Bonferroni correction, and detailed results are provided in Supplementary Table S2

### Logistic regression analysis

To further investigate the association between psychiatric disorder subtype and consultation demand for prosthetic treatment of missing teeth, a logistic regression model was constructed using the enter method, with consultation demand for prosthetic treatment of missing teeth (yes/no) as the dependent variable. Univariable analysis showed that organic mental disorders were significantly associated with consultation demand for prosthetic treatment of missing teeth (OR = 3.69, 95% CI: 2.40–5.67, *P <* 0.001). Age quartiles were also significantly associated with consultation demand for prosthetic treatment of missing teeth, and the risk increased progressively across higher age groups (all *P <* 0.001). Sex was not significantly associated with the outcome (*P =* 0.238).

Multivariable logistic regression analysis showed that, after adjustment for sex and age quartiles, organic mental disorders remained significantly associated with consultation demand for prosthetic treatment of missing teeth (aOR = 2.11, 95% CI: 1.08–4.13, *P =* 0.029). Age was an independent associated factor. Compared with the youngest age group (Q1), the odds of consultation demand for prosthetic treatment of missing teeth were significantly higher in the older age groups (Q2: aOR = 15.93, 95% CI: 4.72–53.77; Q3: aOR = 16.60, 95% CI: 4.69–58.70; Q4: aOR = 16.94, 95% CI: 4.49–63.95; all *P <* 0.001). Sex was not significantly associated with the outcome in the multivariable model (*P =* 0.745) (Table 5).

**Table 5:** Logistic regression analysis of organic mental disorders and consultation demand for prosthetic treatment of missing teeth

Variable	Univariable OR (95% CI)	*P*	Multivariable OR (95% CI)	*P*
Organic mental disorders: No (reference)	1.00	–	1.00	–
Organic mental disorders: Yes	3.69 (2.40–5.67)	< 0.001	2.11 (1.08–4.13)	0.029
Sex: Male (reference)	1.00	–	1.00	–
Sex: Female	1.29 (0.85–1.97)	0.238	1.09 (0.66–1.78)	0.745
Age quartiles: Q1 (reference)	1.00	–	1.00	–
Age quartiles: Q2	15.40 (4.60–51.59)	< 0.001	15.93 (4.72–53.77)	< 0.001
Age quartiles: Q3	23.35 (6.88–79.22)	< 0.001	16.60 (4.69–58.70)	< 0.001
Age quartiles: Q4	32.54 (9.73–108.82)	< 0.001	16.94 (4.49–63.95)	< 0.001

The multivariable model was adjusted for sex and age quartiles. Age quartiles were defined as Q1 (≤ 56 years), Q2 (> 56 to ≤ 68 years), Q3 (> 68 to ≤ 78.5 years), and Q4 (> 78.5 years). The reference groups were non-organic mental disorders, male, and age quartile Q1, respectively

*OR* odds ratio, *CI* confidence interval

A sensitivity analysis was further performed by modeling age as a continuous variable instead of age quartiles. In this model, each additional year of age was associated with a 4% increase in the odds of consultation demand for prosthetic treatment of missing teeth (aOR = 1.04, 95% CI: 1.02–1.06, *P <* 0.001). This result was consistent with the age-related association observed in the primary quartile-based model. Although categorizing age into quartiles yielded larger effect sizes, the continuous variable analysis confirmed a significant linear association between age and prosthetic treatment demand. The consistency between the two approaches strengthens confidence in the clinical relevance of age as an important associated factor (Supplementary Table S3).

## Discussion

### Main findings

This study examined oral consultation patterns across psychiatric disorder subtypes and described the profile of oral problems among psychiatric inpatients who received oral consultations during hospitalization. Because the analysis was restricted to patients who received oral consultations during hospitalization, the results describe consultation patterns in this referred subgroup and do not estimate the overall oral health status of all psychiatric inpatients. Because no non-psychiatric inpatient comparison group was included, these findings cannot determine whether the observed consultation needs are specific to psychiatric inpatients or reflect broader patterns among hospitalized patients. Referral practices, including clinician awareness and patient complaints, may also have influenced the observed consultation distributions by affecting which patients were identified and referred for oral evaluation during hospitalization. Nevertheless, characterizing these consultation patterns remains clinically relevant because psychiatric inpatient care often relies on symptom-driven referral pathways, and identifying subtype-specific consultation profiles may help improve oral health awareness and referral prioritization in psychiatric wards. In general, oral consultations were symptom-driven, with referrals typically initiated only after patients developed obvious discomfort. This pattern was similar across most psychiatric disorder subtypes [8, 9]. It is also consistent with previous reports showing poor oral health and low use of dental services among people with mental disorders, with care often sought only after symptoms such as pain become apparent [10].

Across psychiatric disorder subtypes, patients with organic mental disorders more often presented with consultation demand related to prosthetic treatment of missing teeth, whereas patients with schizophrenia were more likely to be referred for inflammatory or painful oral conditions. Age was independently associated with consultation demand for prosthetic treatment of missing teeth, indicating that age should be considered as an important factor when interpreting the observed differences across psychiatric disorder subtypes.

Tooth pain was the most common reason for consultation. One possible explanation is that dental caries and pulp-related disease are often asymptomatic in the early stage, with pain emerging only after inflammation progresses. In addition, inadequate oral self-care and limited access to routine dental care may allow oral problems to accumulate before hospitalization [11]. During hospitalization, patients may also be more likely to report symptoms that are immediately perceptible, further concentrating referrals on painful conditions.

### Differences by mental disorder type

Oral consultation needs and diagnostic patterns varied across psychiatric disorder subtypes. Organic mental disorders, schizophrenia, and mood disorders were the three psychiatric categories with the highest proportions of oral consultations, broadly consistent with the disease composition of psychiatric inpatients in a general hospital setting. Patients with organic mental disorders more commonly presented with tooth loss and consultation demand for prosthetic treatment of missing teeth, suggesting greater needs related to restoration of oral function in this older subgroup. In contrast, patients with schizophrenia more often presented with symptom-driven consultation needs, such as tooth pain and residual roots/residual crowns.

Patients with organic mental disorders showed the highest proportion of consultation demand for prosthetic treatment of missing teeth, and tooth loss was also most frequent in this group [12, 13]. This finding should be interpreted in the context of the older age profile of this subgroup, with a mean age of 79.15 ± 11.27 years. Tooth loss, periodontal disease, residual roots/residual crowns, and prosthetic treatment needs generally accumulate with increasing age, even in the general population [14–16]. In the present study, nearly half of patients with organic mental disorders had primary oral diagnoses related to tooth loss, periodontal disease, or residual roots/residual crowns, suggesting a substantial burden of chronic oral conditions in this subgroup.

In the primary multivariable model, organic mental disorders remained associated with consultation demand for prosthetic treatment of missing teeth after adjustment for sex and age quartiles (aOR = 2.11, 95% CI: 1.08–4.13), although the effect size was smaller than that observed in the univariable analysis. This suggests that the higher prosthetic treatment demand in this group may not be explained solely by demographic differences. However, because cognitive status, functional ability, caregiver support, medication exposure, oral care practices, and previous dental treatment history were not available, this association should be interpreted as a descriptive and exploratory finding. Even so, it highlights a clinically relevant subgroup in which unmet prosthetic needs may warrant greater attention during psychiatric hospitalization.

Several mechanisms may contribute to the accumulation of unmet prosthetic needs in patients with organic mental disorders. Cognitive impairment, impaired communication, reduced daily functioning, and dependence on caregivers may limit patients’ ability to maintain oral hygiene, seek routine dental care, report oral discomfort, or complete prosthetic rehabilitation before hospitalization. Psychiatric symptoms and reduced cooperation may further affect the feasibility, timing, and complexity of prosthetic management during inpatient care.

Age was also an important associated factor. The higher odds observed in older age quartiles are clinically plausible because tooth loss, periodontal disease, residual roots, and masticatory dysfunction commonly increase with age [14–16]. The sensitivity analysis using age as a continuous variable further confirmed a significant linear association between age and consultation demand for prosthetic treatment of missing teeth. Therefore, the large odds ratios in the quartile-based model should be interpreted as reflecting contrasts between age groups rather than direct per-year effects. In clinical practice, prosthetic treatment decisions for patients with organic mental disorders should consider not only missing teeth, but also cognitive status, cooperation, caregiver support, and expected functional benefit.

Patients with schizophrenia had a relatively high proportion of residual roots/residual crowns, and tooth pain was the most common reason for consultation [17, 18]. This may reflect the combined effects of longer disease duration, reduced self-care ability, and delayed recognition of oral problems [19–21]. Because behavioral factors and disease duration were not available in the present study, these explanations remain tentative.

By contrast, patients with mood disorders and anxiety disorders showed consultation needs and diagnostic profiles that were more similar to those seen in general dental outpatient settings, with pulp-related inflammatory conditions being most prominent [22, 23].

Overall, the differences observed across psychiatric disorder subtypes may reflect the combined influence of disorder-related characteristics and age-related factors. These descriptive findings help identify psychiatric inpatient subgroups with distinct oral consultation profiles and potentially different oral care needs.

### Factors related to oral health in patients with mental disorders

Poor oral health in people with mental disorders is likely to reflect both behavioral and biological influences. On the behavioral side, smoking, alcohol use, high-carbohydrate diets, and neglect of oral hygiene are common in this population [11]. Cognitive and executive dysfunction may further reduce adherence to routine toothbrushing and proactive dental attendance, delaying the detection of oral problems. Low utilization of dental services may also permit oral disease to progress before treatment is sought [7, 8].

From a biological perspective, psychotropic medications can affect oral health by causing xerostomia and reduced salivary secretion. Data based on pharmaceutical company reports indicate that xerostomia was the most frequently reported oral adverse effect among identified psychotropic medications, being reported for approximately 91% of these medications [24]. A recent systematic review reported that tricyclic antidepressants are associated with more severe dry mouth compared with placebo or other drug classes, whereas selective serotonin reuptake inhibitors (SSRIs) are typically linked to milder xerostomia; however, evidence for antipsychotics and many newer psychotropic medications remains limited [5]. Reduced salivary flow may increase the risk of dental caries, periodontal disease, and oral infections. In addition, chronic psychological stress and neuroendocrine alterations may affect oral tissues through inflammatory or immune pathways. Previous studies have reported that individuals with severe mental illness often have poor oral self-care and a higher burden of oral disease [11], which aligns with the findings of the present study.

In addition to behavioral and medication-related factors, age and functional status may influence oral consultation needs in psychiatric inpatients. In the present study, older age was consistently associated with consultation demand for prosthetic treatment of missing teeth, whereas sex was not significantly associated with this outcome. However, unmeasured factors, including psychotropic medication exposure, duration and severity of psychiatric illness, functional status, and self-care ability, may have influenced both psychiatric diagnosis and oral treatment needs.

### Clinical implications

In this psychiatric inpatient setting, oral consultations were mainly triggered by obvious symptoms such as pain, suggesting that some oral problems may remain unrecognized until they become clinically apparent. This symptom-driven referral pattern may lead to under-recognition of chronic or functional oral conditions at an earlier stage.

Consultation needs and diagnostic patterns differed across psychiatric disorder subtypes. In particular, organic mental disorders were associated with consultation demand for prosthetic treatment of missing teeth in the primary quartile-based model, and older age was consistently associated with this outcome across the main and sensitivity analyses. Clinical assessment should therefore take both psychiatric disorder subtype and age into account.

**Fig. 2:**
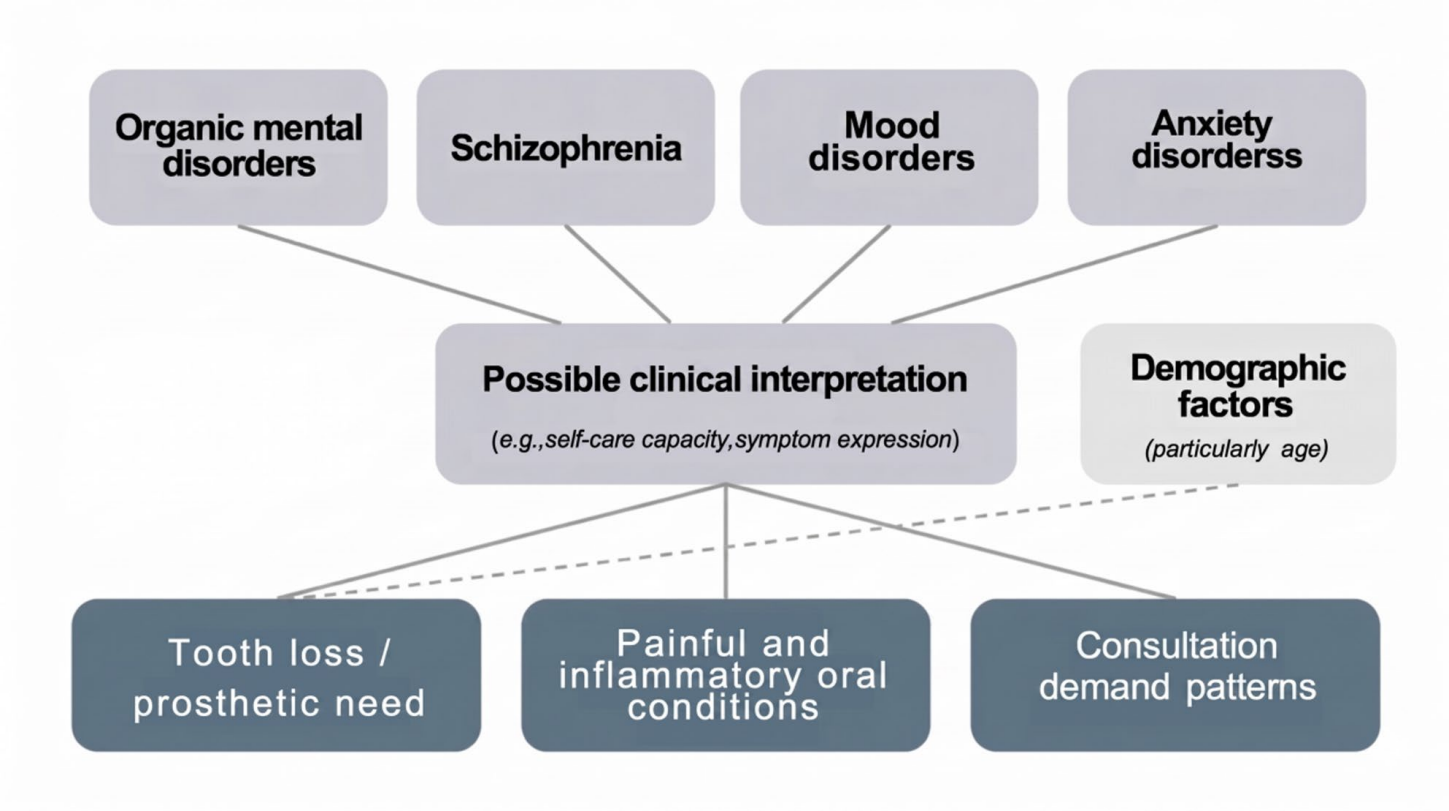
Schematic summary of the main findings of the study. This figure summarizes the study population, overall oral consultation profile, subtype-specific patterns, statistical findings, and multivariable results. The figure is intended for descriptive presentation only and does not imply causal relationships.

Previous studies have suggested that oral health assessment should be strengthened in psychiatric care to improve the recognition of oral problems and reduce unmet treatment needs [8]. A schematic summary of the main findings is shown in Fig. 2. During psychiatric hospitalization, incorporating oral health-related questions or initial oral screening into admission assessment may be helpful [7]. For older patients or those with cognitive impairment, greater attention to tooth loss and masticatory function may be warranted [25, 26], whereas in patients with limited self-care ability, early identification of inflammatory oral conditions may be particularly important. Closer collaboration between psychiatric services and oral health services may improve the detection and management of oral problems [27].

### Limitations and future directions

This study has several limitations. First, only patients who received oral consultations during hospitalization were included, which may have introduced selection bias because the study population was restricted to a subgroup already identified with oral problems or referred for oral evaluation. Accordingly, the findings should be interpreted as reflecting the characteristics and distribution of oral consultations rather than the overall oral health status or prevalence of oral disease among all psychiatric inpatients. In addition, consultation distributions may have been influenced by referral practices, including clinician awareness, service availability, and patient-reported complaints.

Another important limitation is the absence of a non-psychiatric inpatient comparison group. Therefore, the present study cannot determine whether the observed oral consultation needs were specific to psychiatric inpatients or reflected more general patterns among hospitalized patients. The findings should be interpreted as relative differences across psychiatric disorder subtypes within the consulted psychiatric inpatient population, rather than as evidence of excess oral health needs attributable to psychiatric hospitalization or psychiatric illness in general. Future studies should include appropriate comparison groups, such as non-psychiatric inpatients or psychiatric inpatients without oral consultation, to better clarify the specificity and magnitude of oral health needs in this population.

Second, consultation records were analyzed according to a single primary complaint and a single primary diagnosis, which may have simplified clinical reality and underestimated oral comorbidity. Patients may have presented with more than one oral condition at the time of consultation, such as coexisting periodontal disease, tooth loss, and caries-related pain, but only the principal documented item could be analyzed in the present dataset. Accordingly, the reported diagnostic distributions should be interpreted as representing the dominant recorded reason for consultation rather than the full spectrum of oral disease burden in each patient.

Third, some psychiatric subgroups were relatively small, which may have affected the stability of the estimates. In addition, this was a single-center study conducted in a general hospital with an independent psychiatric inpatient campus and access to in-hospital oral consultation services. Although the findings may be informative for general hospitals with psychiatric inpatient wards and similar in-hospital oral consultation pathways, institutional factors, including the availability of oral health services, referral pathways, and clinician awareness of oral problems, may have influenced the observed consultation patterns. Therefore, generalization to other regions, hospitals with different referral protocols, or psychiatric-only hospitals where on-site oral consultation services may be less accessible should be made cautiously. Multicenter studies involving different hospital types and regional settings are needed to further assess the generalizability of these findings.

Because this was a retrospective cross-sectional study based on diagnostic consultation records, the findings should be interpreted as descriptive associations. Nevertheless, they provide useful clinical information on oral consultation patterns and help identify psychiatric inpatient subgroups with potentially higher oral care needs. Although sex and age quartiles were adjusted for in the multivariable analysis, residual confounding remains possible. Important factors such as duration of hospitalization, psychotropic medication type and dose, duration and severity of psychiatric illness, functional status, self-care ability, oral care practices, previous oral disease history, and socioeconomic status were not available in the dataset and may have influenced both referral patterns and the observed association between organic mental disorders and prosthetic treatment needs. Future prospective studies with more comprehensive clinical, behavioral, and medication-related variables are needed to further clarify these relationships and support more targeted oral health assessment and referral strategies in psychiatric inpatient care.

In addition, categorizing age into quartiles may have contributed to the large odds ratios observed for age groups, particularly because the youngest reference group had a relatively low frequency of prosthetic treatment demand. Although quartile-based categorization was used to account for a potentially non-linear relationship between age and prosthetic treatment demand, the sensitivity analysis using age as a continuous variable confirmed a significant age-related association. Future studies with larger samples should further consider flexible non-linear approaches, such as restricted cubic splines, to assess the robustness and shape of age-related associations.

Future studies incorporating multicenter data, appropriate comparison groups, more comprehensive covariate adjustment, and systematic oral screening may provide a more complete assessment of oral health and oral consultation needs across psychiatric disorder subtypes. Prospective studies are also needed to clarify the contributions of behavioral, medication-related, functional, and institutional factors to oral health problems in psychiatric inpatients.

## Conclusions

Oral consultation needs and diagnostic patterns differed across psychiatric disorder subtypes among psychiatric inpatients. In the primary quartile-based multivariable model, organic mental disorders were associated with consultation demand for prosthetic treatment of missing teeth, and age was consistently associated with this outcome across the main and sensitivity analyses. These findings may help inform oral health assessment and referral strategies in psychiatric inpatient care.

## Supplementary Information

Supplementary Material 1.

## Data Availability

The datasets generated and/or analyzed during the current study are available from the corresponding author on reasonable request, subject to institutional and ethical restrictions.

## Abbreviations

ICD-10: International Classification of Diseases, 10th Revision
SD: Standard deviation
OR: Odds ratio
aOR: Adjusted odds ratio
CI: Confidence interval
Q1: First quartile
Q2: Second quartile
Q3: Third quartile
Q4: Fourth quartile
