# Supplementary material for: Oral consultation needs and diagnostic patterns by psychiatric disorder subtype among psychiatric inpatients in a general hospital: a retrospective study
Source: BMC Oral Health. 2026 Jun 2;26:1402. doi: 10.1186/s12903-026-08784-8 (PMC13459250; doi:10.1186/s12903-026-08784-8)
Supplement: Supplementary file 1 [file 12903_2026_8784_MOESM1_ESM.docx]

**Supplementary Material**

*This supplementary material provides detailed post hoc pairwise comparisons and sensitivity analysis supporting the main manuscript.*

**Supplementary Table S1. Pairwise comparisons of oral consultation demands across psychiatric disorder subtypes**

| **Consultation demand** | **Organic mental disorders (n = 165)** | **Schizophrenia (n = 144)** | **Mood disorders (n = 82)** | **Anxiety disorders (n = 17)** | **Other (n = 28)** |
| --- | --- | --- | --- | --- | --- |
| Tooth pain | 49 (29.70)^a^ | 71 (49.31)^a^ | 37 (45.12)^a,b^ | 14 (82.35)^b^ | 14 (50.00)^a,b^ |
| Prosthetic treatment of missing teeth | 77 (46.67)^a^ | 35 (24.31)^b^ | 14 (17.07)^b^ | 0 (0.00)^b^ | 3 (10.71)^b^ |
| Tooth mobility | 11 (6.67)^a^ | 19 (13.19)^a^ | 8 (9.76)^a^ | 1 (5.88)^a^ | 2 (7.14)^a^ |
| Oral mucosal discomfort | 14 (8.48)^a^ | 8 (5.56)^a^ | 9 (10.98)^a^ | 2 (11.76)^a^ | 3 (10.71)^a^ |
| Tooth defect | 4 (2.42)^a^ | 8 (5.56)^a^ | 5 (6.10)^a^ | 0 (0.00)^a^ | 2 (7.14)^a^ |
| Other | 10 (6.06)^a,b^ | 3 (2.08)^b^ | 9 (10.98)^a,b^ | 0 (0.00)^a,b^ | 4 (14.29)^a^ |

**Table note:** Table note: Data are presented as n (%). Superscript letters indicate the results of post hoc pairwise comparisons using Fisher’s exact test with Bonferroni correction. Within the same row, values sharing at least one common superscript letter are not significantly different (adjusted P > 0.05); values with no common superscript letter are significantly different after Bonferroni correction (adjusted P < 0.05).

**Supplementary Table S2. Pairwise comparisons of oral diagnoses across psychiatric disorder subtypes**

| **Oral diagnosis** | **Organic mental disorders** | **Schizophrenia** | **Mood disorders** | **Anxiety disorders** | **Other** |
| --- | --- | --- | --- | --- | --- |
| Tooth loss | 76 (46.06)^a^ | 32 (22.22)^b^ | 14 (17.07)^b^ | 0 (0.00)^b^ | 2 (7.14)^b^ |
| Residual roots/residual crowns | 21 (12.73)^a^ | 40 (27.78)^b^ | 15 (18.29)^a,b^ | 4 (23.53)^a,b^ | 5 (17.86)^a,b^ |
| Pulpitis/apical periodontitis | 20 (12.12)^a^ | 27 (18.75)^a,b^ | 23 (28.05)^b^ | 7 (41.18)^b^ | 10 (35.71)^b^ |
| Periodontal disease | 23 (13.94)^a^ | 25 (17.36)^a^ | 8 (9.76)^a^ | 4 (23.53)^a^ | 3 (10.71)^a^ |
| Oral mucosal disease | 10 (6.06)^a^ | 6 (4.17)^a^ | 7 (8.54)^a^ | 1 (5.88)^a^ | 2 (7.14)^a^ |
| Hard tissue defect | 4 (2.42)^a^ | 11 (7.64)^a^ | 6 (7.32)^a^ | 1 (5.88)^a^ | 2 (7.14)^a^ |
| Other | 11 (6.67)^a,b^ | 3 (2.08)^b^ | 9 (10.98)^a^ | 0 (0.00)^a,b^ | 4 (14.29)^a^ |

**Table note:** Table note: Data are presented as n (%). Superscript letters indicate the results of post hoc pairwise comparisons using Fisher’s exact test with Bonferroni correction. Within the same row, values sharing at least one common superscript letter are not significantly different (adjusted P > 0.05); values with no common superscript letter are significantly different after Bonferroni correction (adjusted P < 0.05).

**Supplementary Table S3. Sensitivity analysis of organic mental disorders and consultation demand for prosthetic treatment of missing teeth using age as a continuous variable**

| **Variable** | **Univariable OR (95% CI)** | **P value** | **Multivariable OR (95% CI)** | **P value** |
| --- | --- | --- | --- | --- |
| Organic mental disorders |  |  |  |  |
| No (reference) | 1.00 | – | 1.00 | – |
| Yes | 3.69 (2.40–5.67) | <0.001 | 1.58 (0.88–2.84) | 0.122 |
| Sex |  |  |  |  |
| Male (reference) | 1.00 | – | 1.00 | – |
| Female | 1.29 (0.85–1.97) | 0.238 | 0.90 (0.56–1.45) | 0.675 |
| Age, years | 1.05 (1.04–1.07) | <0.001 | 1.04 (1.02–1.06) | <0.001 |

**Table note:** OR, odds ratio; CI, confidence interval. The multivariable model included organic mental disorders, sex, and age as a continuous variable.
